# Ivermectin susceptibility and sporontocidal effect in Greater Mekong Subregion *Anopheles*

**DOI:** 10.1186/s12936-017-1923-8

**Published:** 2017-07-07

**Authors:** Kevin C. Kobylinski, Ratawan Ubalee, Alongkot Ponlawat, Chanyapat Nitatsukprasert, Siriporn Phasomkulsolsil, Thanaporn Wattanakul, Joel Tarning, Kesara Na-Bangchang, Patrick W. McCardle, Silas A. Davidson, Jason H. Richardson

**Affiliations:** 1 Department of Entomology, Armed Forces Research Institute of Medical Sciences, 315/6 Rajvithi Road, Bangkok, 10400 Thailand; 2 Entomology Branch, Walter Reed Army Institute of Research, 503 Robert Grant Ave, Silver Spring, MD 20910 USA; 3 Mahidol-Oxford Tropical Medicine Research Unit, Faculty of Tropical Medicine, Mahidol University, 420/6 Rajvithi Road, Bangkok, 10400 Thailand; 4 Centre for Tropical Medicine and Global Health, Nuffield Department of Clinical Medicine, University of Oxford, Old Road Campus, Roosevelt Drive, Oxford, OX3 7FZ UK; 5 Pharmacology and Toxicology Unit, Faculty of Allied Health Sciences, 99 Mu 18 Thammasat University (Rangsit Campus), Klong Luang, Pathumthani 12121 Thailand; 6 Armed Forces Pest Management Board, 2460 Linden Lane, Silver Spring, MD 20910 USA

**Keywords:** Ivermectin, *Anopheles*, *Plasmodium*, Greater Mekong Sub-region

## Abstract

**Background:**

Novel vector control methods that can directly target outdoor malaria transmission are urgently needed in the Greater Mekong Subregion (GMS) to accelerate malaria elimination and artemisinin resistance containment efforts. Ivermectin mass drug administration (MDA) to humans has been shown to effectively kill wild *Anopheles* and suppress malaria transmission in West Africa. Preliminary laboratory investigations were performed to determine ivermectin susceptibility and sporontocidal effect in GMS *Anopheles* malaria vectors coupled with pharmacokinetic models of ivermectin at escalating doses.

**Methods:**

A population-based pharmacokinetic model of ivermectin was developed using pre-existing data from a clinical trial conducted in Thai volunteers at the 200 µg/kg dose. To assess ivermectin susceptibility, various concentrations of ivermectin compound were mixed in human blood meals and blood-fed to *Anopheles dirus*, *Anopheles minimus*, *Anopheles sawadwongporni*, and *Anopheles campestris*. Mosquito survival was monitored daily for 7 days and a non-linear mixed effects model with probit analyses was used to calculate concentrations of ivermectin that killed 50% (LC_50_) of mosquitoes for each species. Blood samples were collected from *Plasmodium vivax* positive patients and offered to mosquitoes with or without ivermectin at the ivermectin LC_25_ or LC_5_ for *An. dirus* and *An. minimus*.

**Results:**

The GMS *Anopheles* displayed a range of susceptibility to ivermectin with species listed from most to least susceptible being *An. minimus* (LC_50_ = 16.3 ng/ml) > *An. campestris* (LC_50_ = 26.4 ng/ml) = *An. sawadwongporni* (LC_50_ = 26.9 ng/ml) > *An. dirus* (LC_50_ = 55.6 ng/ml). Mosquito survivorship results, the pharmacokinetic model, and extensive safety data indicated that ivermectin 400 µg/kg is the ideal minimal dose for MDA in the GMS for malaria parasite transmission control. Ivermectin compound was sporontocidal to *P. vivax* in both *An. dirus* and *An. minimus* at the LC_25_ and LC_5_ concentrations.

**Conclusions:**

Ivermectin is lethal to dominant GMS *Anopheles* malaria vectors and inhibits sporogony of *P. vivax* at safe human relevant concentrations. The data suggest that ivermectin MDA has potential in the GMS as a vector and transmission blocking control tool to aid malaria elimination efforts.

**Electronic supplementary material:**

The online version of this article (doi:10.1186/s12936-017-1923-8) contains supplementary material, which is available to authorized users.

## Background

Artemisinin-resistant *Plasmodium falciparum* isolates have been identified in the Greater Mekong Subregion (GMS) which threatens to undermine malaria elimination goals [1]. The primary GMS *Anopheles* vectors of *Plasmodium* frequently feed outdoors and before people go to sleep [2, 3], rendering classical vector control measures such as insecticide-treated bed nets and indoor residual spraying with insecticides less effective. There are currently no vector control tools in the GMS that specifically target outdoor feeding *Anopheles* which is a major impediment for malaria vector control in the region. Novel vector control tools that target outdoor malaria transmission are urgently needed to support artemisinin resistance containment and malaria elimination efforts in the GMS.

Ivermectin has been shown to be lethal to over a dozen *Anopheles* species worldwide [4]. Ivermectin mass drug administration (MDA) has been suggested as a possible malaria parasite transmission control tool as it directly targets the vector at the point of human blood feeding. It is one of the few vector control measures that targets outdoor malaria transmission. Laboratory studies [5, 6], clinical trials [7–9], and field studies [10, 11] demonstrate that ivermectin is lethal to *Anopheles gambiae* at human relevant concentrations. Ivermectin MDA campaigns in Senegal, Liberia, and Burkina Faso have demonstrated that ivermectin can suppress *P. falciparum* transmission by wild *An. gambiae* s.l. [11, 12]. In addition to direct mosquito-lethal effects, ivermectin suppresses development of *P. falciparum* in *An. gambiae* [13, 14].

*Plasmodium* transmission in the GMS is complex with numerous *Anopheles* species serving as primary and secondary vectors throughout the region. *Anopheles dirus* s.s., found in the GMS east of Myanmar, is a primary malaria vector in forested areas and feeds predominantly outdoors on humans [2, 15]. *Anopheles minimus* s.s., found throughout the GMS and parts of mainland Asia, is a primary malaria vector outside of forested areas, feeds both indoors and outdoors, and displays a variable feeding preference on humans and cattle across its range [3, 16]. There are several secondary malaria vectors that may facilitate malaria transmission in the GMS. *Anopheles sawadwongporni*, a member of the *Anopheles maculatus* group, has been incriminated as a malaria vector in Thailand [17] and *Anopheles campestris*, predominantly found in rice paddies throughout the GMS is suspected to be a secondary malaria vector in Thailand [18].

Ivermectin MDA campaigns in West Africa using a 150–200 µg/kg dose were shown to be effective at reducing *P. falciparum* transmission by *An. gambiae* [11, 12]. However, not all *Anopheles* may be equally susceptible to ivermectin [4]. This implies that higher doses of ivermectin may be required during MDA to effectively target all *Anopheles* in a given region. Doses of ivermectin up to 2000 µg/kg were safe and well tolerated in healthy volunteers [19], which is ten times the amount approved for strongyloidiasis treatment in Thailand [20]. The ivermectin dose of 800 µg/kg has been assessed in onchocerciasis-infected patients in an extended trial in Ghana [21, 22] and repeatedly every 3 months in a trial in Cameroon [23]. Adverse events in the ivermectin 800 µg/kg trials may be correlated with the immune response to dead microfilariae and not necessarily linked directly to ivermectin treatment. Trials in healthy and malaria infected patients without concomitant onchocerciasis infection at the ivermectin 800 μg/kg dose are warranted. The lack of adverse events in healthy volunteers at up to ivermectin doses of 2000 μg/kg [19] would support the notion that the adverse events observed in Ghana [21, 22] and Cameroon [23] trials may be linked to the death and clearance of *Onchocerca volvulus* parasites and not ivermectin toxicity. Several clinical trials have investigated the safety and tolerability of ivermectin at 400 and 800 µg/kg, however, to the best of our knowledge, none have assessed the pharmacokinetic properties of ivermectin at these doses, particularly in an Asian population, which is the population of interest in this study.

The mosquito-lethal effects of ivermectin on the GMS malaria vectors *An. dirus* s.s., *An. minimus* s.s., *An. sawadwongporni,* and *An. campestris*, and the sporontocidal effects of ivermectin on *Plasmodium vivax* in *An. dirus* s.s., and *An. minimus* s.s. were investigated. A population-based pharmacokinetic model of ivermectin and simulated concentration–time profiles after 200, 400 and 800 µg/kg doses was developed, and correlated with mosquito survivorship results to rationally select ivermectin doses for MDA use in the GMS.

## Methods

### Pharmacokinetic modelling

Frequent ivermectin plasma concentrations were collected in 23 healthy Thai volunteers after a standard 200 µg/kg dose [24]. Ivermectin concentrations were transformed into their natural logarithms and characterized using nonlinear mixed-effects modelling in NONMEM version.7.3 (Icon Development Solution, Ellicott City, MD, USA). The first-order conditional estimation method with interaction was used throughout model development. Model diagnostics and automation were performed using Xpose version 4.0 [25], Pirana [26] and Pearl-speaks-NONMEM (PsN; version 3.6.0) [27]. The objective function value (OFV; calculated by NONMEM as proportional to −2 × loglikelihood of data) was used to discriminate between hierarchical models. A difference between two models (ΔOFV) of >3.84 and >10.83 were considered statistically significant at a P value of <0.05 and <0.001, respectively. Models were assessed using standard goodness-of-fit and simulation-based diagnostics (i.e. numerical and visual predictive checks using 2000 simulations). The robustness of the final model was evaluated by bootstrapping (n = 1000).

Different disposition models (one-, two- and three-compartment models) and absorption models (first-order and transit models) were evaluated to describe the pharmacokinetic structural model of ivermectin. Pharmacokinetic parameters were assumed to be log-normally distributed and inter-individual variability was implemented as exponential functions. The effect of body size was evaluated using an allometric function (centred on the medium trial weight of 55 kg), scaling clearances with an exponent of ¾ and volumes with an exponent of 1. The influence of other individual covariates including gender, age, haemoglobin, haematocrit, total bilirubin, direct bilirubin, alkaline phosphatase, aspartate aminotransferase, and alanine aminotransferase, serum creatinine, blood urea nitrogen, and albumin level were investigated using a stepwise covariate approach (P value <0.05 and <0.001 for forward and backward addition and deletion, respectively). The developed final population pharmacokinetic model of ivermectin was used to simulate population mean ivermectin pharmacokinetic concentration–time profiles after a single oral dose of 200, 400 and 800 µg/kg using Berkeley Madonna [28].

### Mosquitoes

All mosquitoes were reared at the Armed Forces Research Institute of Medical Sciences Department of Entomology in Bangkok, Thailand. *Anopheles dirus* s.s., *An. minimus* s.s., *An. sawadwongporni*, and *An. campestris* were produced as described previously [29]. Adult mosquitoes used for experiments were provided 10% sucrose solution ad libitum. Mosquitoes were reared at 25 ± 2 °C and 80 ± 10% relative humidity, and 12 h light:12 h dark photoperiod. Mosquitoes were between 5 and 8 days post emergence at time of first blood feed, and mosquitoes were sugar starved with access to water from 12 to 18 h prior to their first blood meal.

### Drug

Powdered formulation of ivermectin compound was obtained from Sigma-Aldrich (St. Louis, USA). Ivermectin was dissolved in dimethylsulfoxide (DMSO) to concentrations of 10 mg/ml and 20 µl aliquots were frozen at −20 °C. Ivermectin was thawed and serial dilutions were made in phosphate buffered saline (PBS) and 10 μl was added to 990 μl of blood to reach final concentration desired for mosquito membrane feeding assays. Control blood meals consisted of previously frozen DMSO diluted in PBS to match the ratio of DMSO and PBS fed to mosquitoes in the ivermectin-containing blood meals.

### Blood and serum

Pooled whole blood collected in 350-ml citrate phosphate dextrose adenine (CPDA-1) anticoagulant bags (National Blood Centre, Thai Red Cross Society, Bangkok, Thailand) was used to maintain colonies and perform ivermectin lethal concentration experiments. Blood was never more than 2 weeks post collection at the time of mosquito blood feeds. *Plasmodium vivax*-infected blood was drawn from individual volunteers into 10-ml sodium heparin tubes (NH) (158 USP units, BD Vacutainer, Franklin Lakes, NJ, USA) for the ivermectin sporogony experiments. Serum (type AB+) from malaria-naïve persons (Interstate Blood Bank Inc, Memphis, TN, USA) was processed and stored frozen at −20 °C until ready for use in ivermectin sporogony experiments.

### Ivermectin lethal concentration calculations

*Anopheles dirus*, *An. minimus*, *An. sawadwongporni,* and *An. campestris* were blood fed multiple concentrations of ivermectin to determine the lethal concentration that killed 50% (LC_50_), 25% (LC_25_) and 5% (LC_5_) of the mosquitoes at day 7 post blood meal, following previous methods [5, 13]. Control blood meals consisted of DMSO diluted in PBS to match the concentration found in the highest ivermectin treatment group in each replicate. After blood feeding via a membrane feeder, unfed mosquitoes were removed and discarded. Blood-fed mosquitoes were held in 3.5-l plastic containers with access to 10% sucrose and kept in an incubator at 25 ± 1 °C and 70 ± 10% relative humidity, on a 12 h light:12 h dark photoperiod. Mosquito survivorship was monitored for 7 days, every 24 h dead mosquitoes were removed and recorded, and on day 7 all remaining mosquitoes were frozen and counted as alive.

The time above the LC_50_-values for different species of mosquitoes was determined from the simulated pharmacokinetic concentration–time profiles after a single oral dose of 200, 400 and 800 µg/kg, as described above.

### Effect of ivermectin on *Plasmodium vivax* sporogony

Previously it was demonstrated that the ivermectin LC_25_ inhibited the development of *P. falciparum* in *An. gambiae* [13], and a similar experimental design was repeated here. Mosquitoes reared in Bangkok were transported to a field laboratory at the Mae Sot Malaria Medical Clinic in Mae Sot, Tak Province. *Plasmodium vivax*-infected blood was collected from malaria-infected patients reporting to malaria clinics operated by the Thailand Ministry of Public Health in Tak Province (protocol WRAIR#1949A). Patient plasma was removed and replaced by centrifuging blood samples at 3000 rpm for 5 min, plasma removed and blood washed with RPMI twice, and malaria-naïve AB+ serum was added at a 50:50 ratio [30]. One millilitre of blood was fed to each group of 100 mosquitoes in 0.5-l cardboard containers and unfed mosquitoes were removed. Mosquitoes were maintained on 10% sucrose ad libitum at the Mae Sot field insectary (25 ± 1 °C and 70 ± 10% relative humidity, light 12 h:12 h dark photoperiod). Once infected with *P. vivax*, blood-fed mosquitoes were securely transported back to Bangkok and maintained in a separate insectary (25 ± 1 °C and 70 ± 10% relative humidity, light 12 h:12 h dark photoperiod).

**Fig. 1:**
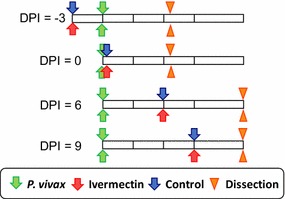
Experimental design for determining the effect of ivermectin against *Plasmodium vivax* in *Anopheles dirus*. Each timeline depicts when ivermectin (*red arrow*), control (*blue arrow*), and *P. vivax* (*green*) blood meals were offered to mosquitoes and when dissections (*orange triangle*) occurred

For *An. dirus*, four experimental feeding schedules performed where ivermectin LC_25_ and LC_5_ was blood fed concomitantly with *P. vivax* at day post infection zero (DPI 0), ivermectin LC_5_ three  days before (DPI −3) *P. vivax*, and ivermectin LC_25_ six (DPI 6) or nine (DPI 9) after *P. vivax* (Fig. 1). For *An. minimus* only one experimental feeding schedule was performed when ivermectin LC_25_ and LC_5_ was blood fed concomitantly with *P. vivax* (DPI 0). For each mosquito species and feeding schedule there was an ivermectin (LC_25_ or LC_5_) and control group, which had matching concentrations of DMSO and PBS. When mosquitoes were fed ivermectin or control meals at DPI −3, 6 or 9, uninfected donor blood at a 50% haematocrit was used for the blood feed. Unfed mosquitoes were removed from the feeding containers after each blood meal. Mosquitoes from the DPI 0 and DPI −3 feeding schedules were dissected 7 days post parasite ingestion to enumerate oocysts. Midguts were dissected with minuten pins into saline on a microscope slide and stained with 0.4% mercurochrome and viewed at 40× magnification with a compound microscope to determine oocyst prevalence and intensity. Approximately 25 mosquitoes were dissected from each control and treatment group at each dissection time point. Dissectors were blinded to treatment groups. Due to lack of initial mosquito blood feeding and mosquitoes lost to experimental mortality and oocyst dissections at day 7 post parasite feeding, not enough mosquitoes remained at day 14 post parasite feeding to determine sporozoite prevalence for the DPI −3 and 0 experiments. Mosquitoes from the DPI 6 and 9 experiments were dissected 14 days post parasite ingestion to determine sporozoite prevalence. Salivary glands were dissected with minuten pins into saline on a microscope slide and viewed at 40× magnification to determine sporozoite prevalence.

### Effect of *Plasmodium vivax* infection on *Anopheles dirus* survivorship response to ivermectin

Previously it was shown that *An. gambiae* infected with *P. falciparum* were slightly but significantly more susceptible to an ivermectin blood meal at day 14 post parasite ingestion [13]. *Anopheles dirus* were fed a *P. vivax* blood meal or a non-infectious blood meal prepared from uninfected donor blood and serum was replaced as described above. Both blood meals were spiked with ivermectin at the LC_25_. After blood feeding, 50 blood fed females from each treatment group were transferred to a new 0.5-l cardboard carton. Mosquito survivorship was monitored daily and dead mosquitoes were removed from the containers until day 14 post blood meal. Any live mosquitoes at day 14 were frozen and counted as alive. A separate group of mosquitoes were fed a *P. vivax* control blood meal to ensure that the *P. vivax* sample was infectious to mosquitoes and only isolates that successfully infected at least 80% of *An. dirus* were included in the results.

### Statistical analysis

A non-linear mixed effects model with probit analysis was used to calculate LC_50_, LC_25_ and LC_5_ values with Statistical Analysis Software (SAS Institute Inc, Cary, NC, USA) as described previously [5]. Hazard ratios for mosquito mortality at day 7 post blood meal were calculated using Poisson regression analysis with STATA version 12.1 (StataCorp LLC, College Station, TX, USA). Oocyst and sporozoite prevalence (i.e. proportion of infected mosquitoes) was compared by Fishers Exact test. Oocyst intensity (i.e. number of oocysts per infected mosquito) was compared by the Mann–Whitney U test. To determine if *P. vivax* infection altered mosquito survivorship post-ivermectin ingestion survival data were analysed by the Mantel–Cox method. The Fishers Exact, Mann–Whitney U, and survival analysis data were analysed with Prism 7 (GraphPad Software Inc, San Diego, CA, USA).

## Results

### Pharmacokinetic modelling

**Fig. 2:**
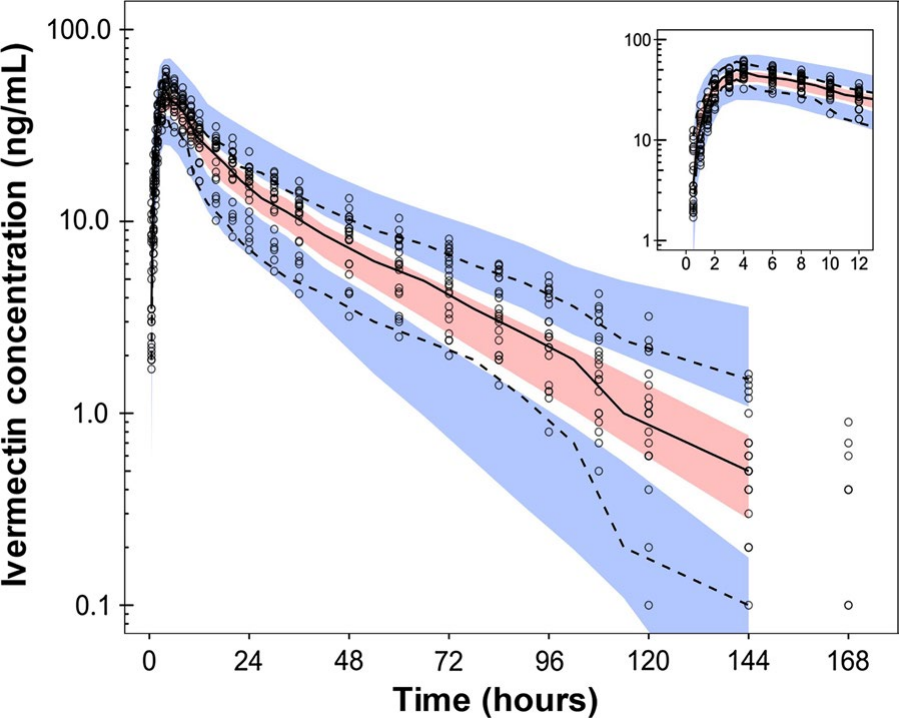
Visual predictive check of final population pharmacokinetic model of ivermectin in healthy volunteers. *Open circles* represent observed concentrations; *solid* and *dashed lines* represent the 5th, 50th, and 95th percentiles of the observed data; *shaded areas* represent the 95% confidence intervals of the simulated 5th, 50th and 95th percentiles (n = 2000)

**Fig. 3:**
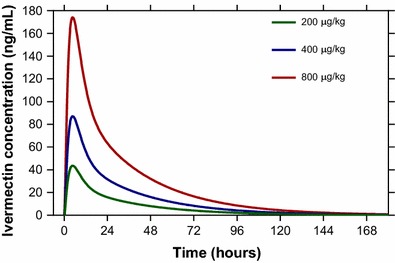
Simulation population mean pharmacokinetic profiles of ivermectin at single oral doses of 200, 400 and 800 µg/kg, based on final population pharmacokinetic model

A total of 534 venous blood samples were collected from 23 healthy volunteers (12 males and 11 females) for up to 7 days after a single oral dose of 200 µg/kg of ivermectin. There were no observations reported to be below the lower limit of quantification. Observed ivermectin concentrations were best described by a two-compartment disposition model, with no further improvement of an additional disposition compartment (∆OFV = 0). A flexible transit absorption model (n = 2) improved the model fit substantially compared to the first-order absorption (∆OFV = −378). The implementation of body weight as an allometric function had a negligible impact on model fit (∆OFV = 0.516), but it was retained in the final model based on the strong biological prior. Implementing body weight as a covariate in the model also allowed for translation and interpretation to other study populations. No additional covariates had a significant impact on the model fit. The final model showed satisfactory goodness of fits (Additional file 1: Figure S1) and simulation-based predictive performance (Fig. 2). A numerical predictive check resulted in 2.62% (95% CI 0.37–6.18%) and 0.56% (95% CI 0.56–5.43%) of ivermectin observations below and above the simulated 95% prediction interval, respectively. Ivermectin population pharmacokinetic parameter estimates and secondary parameters from final model are summarized in Table 1. The simulated concentration–time profiles after a single oral dose of 200, 400 and 800 µg/kg of ivermectin are illustrated in Fig. 3.

**Table 1:** Parameter estimates from final population pharmacokinetic model of ivermectin in healthy volunteers

Parameter	Population estimates (%RSE)^b^	95% CI^b^	IIV [%CV] (%RSE)^b^	95% CI^b^
F	1 (fixed)	–	10.7 (42.9)	4.82–14.1
k_a_ (h^−1^)	0.317 (3.97)	0.296–0.343	–	–
MTT (h)	0.496 (17.1)	0.350–0.679	56.1 (36.2)	31.9–77.4
CL/F (l/h)	9.02 (5.49)	8.08–10.1	23.1 (25.2)	15.3–27.5
V_C_/F (l)	115 (4.38)	106–125	–	–
Q/F (l/h)	16.2 (5.34)	14.7–18.1	–	–
V_P_/F (l)	157 (6.59)	139–178	22.8 (36.0)	13.5–29.9
σ	0.0361	0.0263–0.0463	–	–
Secondary parameters^c^				
Terminal half-life (h)	25.0 (23.7–29.5)			
AUC_0–168_ (ng × h/ml)	1331 (919–1406)			
C_max_ (ng/ml)	45.7 (40.3–46.9)			
T_max_ (h)	4.76 (4.67–5.04)			

*F* relative bioavailability, *k*_*a*_ absorption rate constant, *MTT* mean transit absorption time, *CL/F* apparent oral elimination clearance, *V*_*C*_ */F* apparent volume of distribution of central compartment, *Q/F* apparent inter-compartmental clearance, *V*_*P*_ */F* apparent volume of distribution of peripheral compartment, *σ* variance of the residual variability

^a^Population mean values and inter-individual variability (IIV) were estimated by NONMEM. The coefficient of variation (%CV) for IIV was calculated as $100 \times \sqrt {e^{estimate} - 1}$

^b^The relative standard error (%RSE) was calculated as $100 \times \left( {\frac{\text{SD}}{{{\text{Mean}}\;{\text{value}}}}} \right)$ from the non-parametric bootstrap results (n = 1000). The 95% confidence interval (95% CI) is presented as the 2.5–97.5 percentiles of the bootstrap estimates

^c^Post hoc parameter estimates from final pharmacokinetic model of ivermectin presented as median (interquartile range)

### Ivermectin lethal concentration calculations

**Fig. 4:**
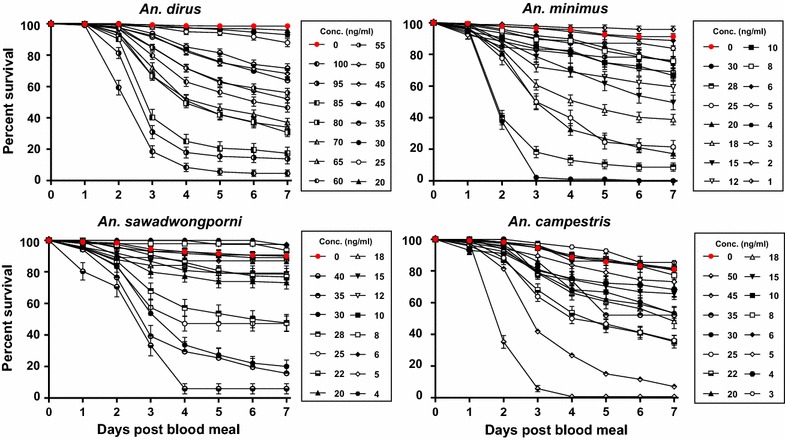
*Anopheles* survival post ingestion of ivermectin compound by day. *Boxed legends* represent the concentrations of ivermectin imbibed by each species. Not all concentrations included in the lethal concentration analyses are displayed here. Each *line* represents 1–6 replicates with standard error

A total of 11,622 mosquitoes were used to calculate the concentration of ivermectin that killed: *An. dirus* (reps = 6, n = 5029), *An. minimus* (reps = 6, n = 2376), *An. sawadwongporni* (reps = 4, n = 1446), and *An. campestris* (reps = 4, n = 2786). Ivermectin compound at human-relevant concentrations reduced the survivorship of all GMS *Anopheles* species investigated (Fig. 4). The GMS *Anopheles* displayed a range of susceptibility to ivermectin with species listed from most to least susceptible being *An. minimus* > *An. campestris* = *An. sawadwongporni* > *An. dirus* (Table 2). The amount of time that ivermectin compound was predicted to be above each *Anopheles* species 7-day-LC_50_ concentration in human plasma for each 200, 400 and 800 µg/kg dose is reported in Table 3. For *An. dirus* all ivermectin concentrations had a significantly increased hazard of mortality compared to the control group; for *An. minimus* all ivermectin concentrations had significantly increased hazard of mortality compared to the control group except for 6, 3 and 2 ng/ml; for *An. sawadwongporni* all ivermectin concentrations had significantly increased hazard of mortality compared to the control group except for 12, 10, 8, 6, 5 and 4 ng/ml; and for *An. campestris* all ivermectin concentrations had significantly increased hazard of mortality compared to the control group except for 10, 6, 5 and 3 ng/ml (Additional file 1: Table S1).

**Table 2:** Lethal concentration values by *Anopheles* species at 7 days post blood meal

	LC_50_	95% FL*	LC_25_	95% FL	LC_5_	95% FL
*An. dirus*	55.6	[52.3–59.1]	38.1	[34.4–41.3]	22.1	[18.1–25.6]
*An. minimus*	16.3	[11.6–19.4]	11.3	[5.7–14.5]	6.7	[1.9–10.1]
*An. sawadwongporni*	26.9	[24.8–28.8]	21.8	[18.6–23.8]	16.1	[11.9–18.8]
*An. campestris*	26.4	[21.9–30.5]	18.9	[13.9–22.6]	11.7	[6.9–15.5]

*FL 95% fiducial limits

**Table 3:** Time (in days) above each *Anopheles* species 7-day-LC_50_ by dose as predicted by the pharmacokinetic model

Dose	200 µg/kg	400 µg/kg	800 µg/kg
*An. dirus*	n/a	0.4	1.1
*An. minimus*	0.9	1.9	3
*An. sawadwongporni*	0.4	1.2	2.2
*An. campestris*	0.5	1.2	2.3

### Effect of ivermectin on *Plasmodium vivax* sporogony

**Fig. 5:**
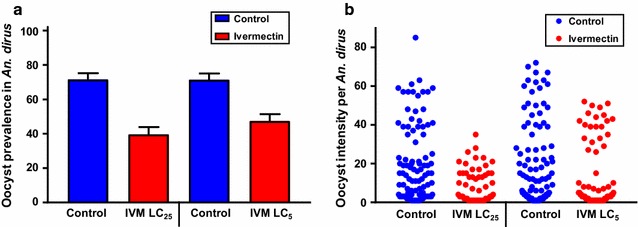
*Plasmodium vivax* oocyst prevalence (**a**) and intensity (**b**) in *Anopheles dirus* when ivermectin co-ingested with parasites. *Plasmodium vivax* oocyst prevalence (**a**) and intensity (**b**) in *An. dirus* when ivermectin LC_25_ (38.1 ng/ml) and LC_5_ (22.1 ng/ml) co-ingested with parasites at DPI 0. Oocyst prevalence was significantly reduced at the LC_25_ and LC_5_ concentrations as determined by the Fishers Exact test. Oocyst intensity was significantly reduced at the LC_25_ and LC_5_ concentrations as determined by the by the Mann–Whitney U test. Prevalence *error bars* represent standard error

**Fig. 6:**
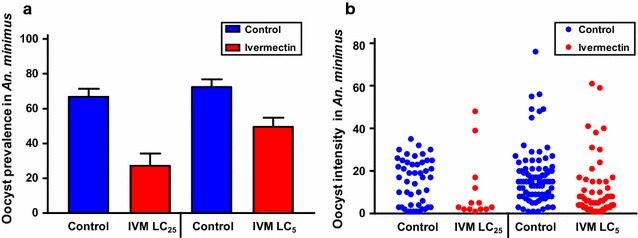
*Plasmodium vivax* oocyst prevalence (**a**) and intensity (**b**) in *Anopheles minimus* when ivermectin co-ingested with parasites. *Plasmodium vivax* oocyst prevalence (**a**) and intensity (**b**) in *An. minimus* when ivermectin LC_25_ (11.3 ng/ml) and LC_5_ (6.7 ng/ml) co-ingested with parasites at DPI 0. Oocyst prevalence was significantly reduced at the LC_25_ and LC_5_ concentrations as determined by the Fishers Exact test. Oocyst intensity was significantly reduced at the LC_5_ but not the LC_25_ concentration as determined by the by the Mann–Whitney U test. Prevalence *error bars* represent standard error

When co-ingested at DPI 0, ivermectin reduced the development of *P. vivax* in both *An. dirus* and *An. minimus*. In *An. dirus* the *P. vivax* oocyst prevalence was reduced at DPI 0 at the ivermectin LC_25_ by 44.7% (χ^2^ = 29.52, *P* < 0.0001, reps = 6, n = 285) and LC_5_ reduced by 33.6% (χ^2^ = 17.9, *P* < 0.0001, reps = 6, n = 300) (Fig. 5a) and mean oocyst intensity was reduced at the LC_25_ by 51.2% (*P* = 0.0022, n = 156) and LC_5_ by 24.7% (*P* = 0.0389, n = 178) (Fig. 5b). In *An. minimus* the *P. vivax* oocyst prevalence was reduced at DPI 0 at the ivermectin LC_25_ by 58.8% (χ^2^ = 21.72, *P* < 0.0001, reps = 5, n = 172) and LC_5_ by 31.3% (χ^2^ = 12.93, *P* = 0.0004, reps = 5, n = 235) (Fig. 6a) and mean oocyst intensity was reduced at the LC_25_ by 28.5% (*P* = 0.1018, n = 60) and LC_5_ by 35.3% (*P* < 0.0001, n = 139) (Fig. 6b). Note, the lack of significance for oocyst intensity for *An. minimus* at the ivermectin LC_25_ is due to mosquito mortality and sporontocidal effect reducing oocyst prevalence contributing to too few mosquitoes to dissect in the treatment group to determine significance.

**Fig. 7:**
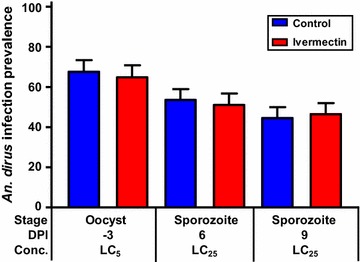
*Plasmodium vivax* infection prevalence in *Anopheles dirus* when ivermectin ingested at DPI −3, 6, and 9. *Plasmodium vivax* infection prevalence in *An. dirus* when ivermectin LC_5_ (22.1 ng/ml) ingested at DPI −3 and LC_25_ (38.1 ng/ml) ingested at DPI 6 and 9. Oocyst prevalence was not significantly reduced at the DPI −3, 6, or 9 time points as determined by the Fishers Exact test. Prevalence *error bars* represent standard error

However, ivermectin did not significantly alter *P. vivax* infection in *An. dirus* when given at DPI −3, 6 or 9. *Plasmodium vivax* oocyst prevalence decreased slightly in *An. dirus* at DPI −3 at the LC_5_ by 3.9% (χ^2^ = 0.12, *P* = 0.4313, reps = 3, n = 150) (Fig. 7) and oocyst intensity increased by 24.2% (*P* = 0.4405, n = 100) (Additional file 1: Figure S2). *Plasmodium vivax* sporozoite prevalence in *An. dirus* decreased slightly at DPI 6 at the LC_25_ by 4.5% (χ^2^ = 0.11, *P* = 0.7751, reps = 4, n = 195) and increased slightly at DPI 9 at the LC_25_ by 4.4% (χ^2^ = 0.08, *P* = 0.8872, reps = 4, n = 200) (Fig. 7).

### Effect of *Plasmodium vivax* infection on *Anopheles dirus* survivorship response to ivermectin

**Fig. 8:**
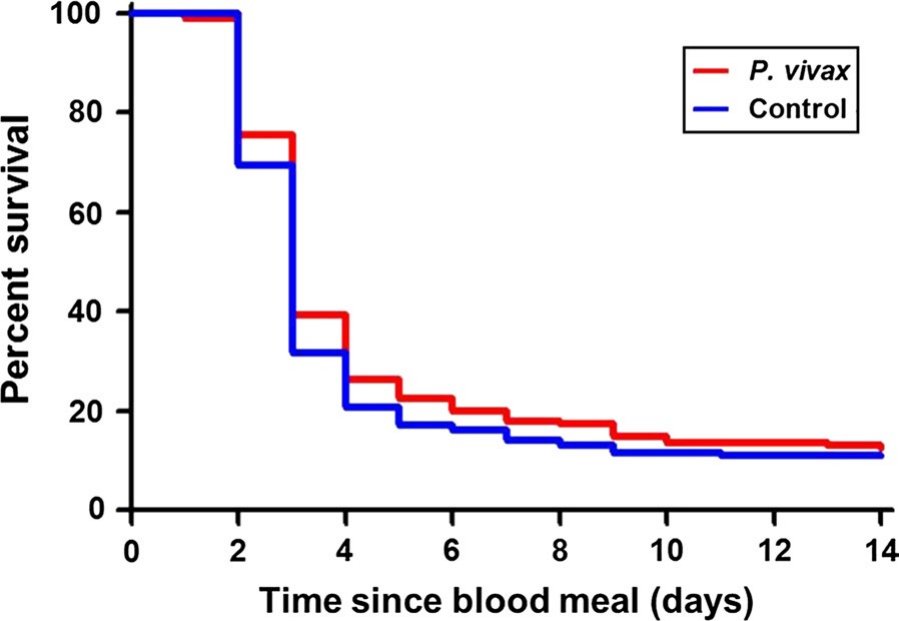
Survivorship of *Anopheles dirus* when ivermectin ingested with and without *Plasmodium vivax*. Survivorship of *An. dirus* when ivermectin LC_25_ (38.1 ng/ml) ingested with and without *P. vivax*. Survivorship between mosquito treatment groups was not significantly different as determined by the Mantel–Cox method

*Plasmodium vivax* infection did not alter survivorship of *An. dirus* that co-ingested parasites and ivermectin LC_25_ on DPI 0 (χ^2^ = 2.83, *P* = 0.0925, reps = 4, n = 400) compared to control mosquitoes that only ingested ivermectin LC_25_ (Fig. 8).

## Discussion

All four *Anopheles* species investigated were susceptible to ivermectin at concentrations predicted to be present in humans following oral administration. This suggests that ivermectin MDA has a potential role in malaria elimination in the GMS as it is a novel vector control tool that could directly combat outdoor malaria transmission. *Anopheles dirus* and *An. minimus* are the two most important primary vectors of malaria in the GMS while *An. campestris* and *An. sawadwongporni* are potential secondary vectors [31]. Since *An. dirus* is arguably the most important GMS malaria vector it is critical that ivermectin MDA deliver a dose of ivermectin high enough to control this vector. Population peak ivermectin concentrations after a single oral dose of 200 µg/kg did not reach the 7-day-LC_50_ of *An. dirus*. However, pharmacokinetic modelling and simulation presented here suggest that a single oral ivermectin dose of 400 or 800 µg/kg results in concentrations that surpasses the ivermectin 7-day-LC_50_ for *An. dirus* (Table 3). Based on the *An. dirus* in vitro survivorship results, pharmacokinetic model output and extensive safety data discussed below, the ivermectin 400 µg/kg dose appears to be the ideal minimal dose to administer during MDA for malaria control in the GMS.

The simulated time above the *An. dirus* ivermectin 7-day-LC_50_ at ivermectin doses of 400 µg/kg (0.4 days) or 800 µg/kg (1.1 days) would not seem to deliver a substantial mosquito-killing window following ivermectin MDA. However, it should be noted that the 200 µg/kg dose would only provide a simulated 0.6 days above the *An. gambiae* 5-day-LC_50_ value of 22.4 ng/ml [5], and yet ivermectin MDAs in Senegal at doses of 150 µg/kg substantially reduced *An. gambiae* 5-day survivorship by 43.6% for up to 6 days post MDA [10]. Furthermore, the pharmacokinetic model predicts that only 0.432 ng/ml of ivermectin parent compound would be present in a typical patient of 55 kg body weight 6 days post MDA at the 150 µg/kg dose, which is well below the concentration capable of killing *An. gambiae*. This clearly illustrates that in vitro mosquito membrane feeds and pharmacokinetic predictions of parent compound likely underestimate the full mosquito-lethal potential of ivermectin-treated humans. One possible explanation may be that ivermectin produces active mosquito-lethal in vivo metabolites with different pharmacokinetic properties that extend the duration of mosquito-lethal effect beyond that of the parent compound. Human liver microsomes have been used to characterize some of the ivermectin metabolites [32]. A small (n = 4) mass balance study in humans determined that mean peak plasma concentration of metabolites was 2.5-fold greater than that of the parent compound and the effective half-lives of the metabolites was approximately 2.9 days while the parent compound half-life was 11.8 h [33]. Further attention to the characterization of ivermectin mosquito-lethal metabolites is warranted, especially in light of the novel long-lasting ivermectin formulations in development for human use [34–36].

The final population pharmacokinetic model developed in this study described ivermectin concentrations in healthy volunteers satisfactorily. Overall parameter estimates were similar to that reported previously in healthy volunteers and patients with onchocerciasis [37, 38]. Simulation-based diagnostics demonstrated a good predictive performance, which suggests that the developed model is suitable to use for simulations. However, the model was developed on data from a single oral dose of 200 µg/kg and extrapolations beyond that (i.e. simulations of 400 and 800 µg/kg doses) assumes that ivermectin shows dose linearity at this dosing range [19]. Body weight, implemented as allometric function, produces a biologically plausible covariate relationship [39, 40] between ivermectin exposure and body weight so that the developed pharmacokinetic model can be used to simulate other populations at risk, such as children. However, the pharmacokinetic properties of a drug can be very different in children and adults due to the rapid change in body size, organ function, body composition, and enzyme maturation, which occur during the early years of life. Prospective clinical trials are urgently needed in children since there are currently no pharmacokinetic assessments of ivermectin in children and adolescents below 17 years of age. A previous study [8] reported that female participants and participants with higher body mass index (BMI) had higher day-7 ivermectin concentrations. However, BMI was not found to be a significant covariate on any pharmacokinetic parameters in the current analysis, perhaps due to the relatively narrow range of BMI (17.8–22.8 kg/m^2^) studied here. Simulation of ivermectin concentration–time profiles at different dosing regimens were also performed in a previous study [41]. The authors obtained pharmacokinetic parameters from the literature, based on American healthy volunteers and assuming a 30% inter-individual variability in each parameter. Their simulated concentration–time profile after a single dose of 800 µg/kg of ivermectin resulted in an average peak concentration of 108.1 ng/ml [41], which is somewhat lower than the value simulated using the model developed here (174 ng/ml).

The ivermectin dose of 400 µg/kg has been investigated in thousands of adults and children, in healthy and infected persons (e.g. lymphatic filariasis, onchocerciasis, loaisis, ascariasis, trichuriasis, hookworm and lice), in more than 20 clinical trials in ten countries, including India [42], Cameroon [23], Ghana [43], Gabon [44], Sri Lanka [45–47], Mali [48], Papua New Guinea [49, 50], French Polynesia [51–58], Brazil [59–62], Haiti [63, 64], and France [65]. Repeated ivermectin administration at doses of 400 µg/kg was safe in two trials in adults who were treated every two weeks for 12 weeks in Sri Lanka [45] and Brazil [61]. The ivermectin dose of 400 µg/kg was deemed safe enough to perform several rounds of MDA to thousands of people in India [42], Cameroon [23], Papua New Guinea [49], and French Polynesia [56, 57] with minimal adverse events reported. Ramaiah and colleagues led the largest MDA trial of ivermectin 400 µg/kg study to date, wherein five entire villages, roughly 10,000 people, including children and adults of both gender, were treated by MDA nine times over an 11-year period. The 400 µg/kg ivermectin dose is now recommended for lymphatic filariasis when twice yearly ivermectin 200 µg/kg MDA cannot logistically be performed [66]. This extensive safety data should justify the use of ivermectin 400 µg/kg for MDA in the GMS.

The sporogony experiments conducted here clearly indicate a sporontocidal effect of ivermectin against *P. vivax* in both *An. dirus* and *An. minimus* (Figs. 5, 6). Both *P. vivax* oocyst prevalence and intensity were significantly reduced when ivermectin compound at the LC_25_ and LC_5_ when ingested concomitantly (DPI 0) with parasites in *An. dirus* and *An. minimus*. A previous study found that ivermectin compound significantly reduced *P. falciparum* prevalence in *An. gambiae* when ingested concomitantly (DPI 0) at the ivermectin LC_25_ but not the LC_5_, and had no effect on oocyst intensity at either concentration [13]. A sporontocidal effect would reduce onward transmission from infected persons that received ivermectin MDA while gametocytaemic. This may be more relevant for transmission suppression for *P. vivax* than *P. falciparum* as *P. vivax* gametocytes mature much more rapidly and are therefore present before people become ill enough to seek treatment [67]. These studies only assess the sporontocidal effect of ivermectin in the mosquito, future studies should investigate the potential gametocytocidal action of ivermectin.

There was no effect of ivermectin LC_5_ compound on *P. vivax* oocyst prevalence (Fig. 7) or intensity (Additional file 1: Figure S2) when ingested by *An. dirus* at DPI −3. This is in direct contrast to previous findings which showed ivermectin compound LC_5_ at DPI −3 reduced oocyst prevalence of *P. falciparum* in *An. gambiae* [13]. Ingestion of blood from ivermectin-treated cattle 4 days before ingestion of field isolates of *P. falciparum* from infected patients did not have a sporontocidal effect [68]. Ivermectin LC_25_ compound failed to reduce *P. vivax* sporozoite prevalence in *An. dirus* when ingested at DPI 6 or 9 (Fig. 7), while there was a significant sporontocidal effect at the ivermectin LC_25_ at DPI 6 and 9 for *P. falciparum* in *An. gambiae* [13]. This indicates that ivermectin can have differential sporontocidal impact with different *Anopheles* and *Plasmodium* species combinations.

Previously it was suggested that ivermectin sporontocidal effect may be due to direct effects on the mosquito, specifically ivermectin delaying or altering formation of the peritrophic matrix [13]. Indeed, ivermectin has been shown to delay and alter peritrophic matrix formation in *Aedes aegypti* [69], delay blood meal digestion in *An. gambiae* [5], and upregulates peritrophic matrix gene expression following ivermectin ingestion in *An. gambiae* [70]. However, limited investigations by two other laboratories were unable to identify a sporontocidal effect of ivermectin against *P. falciparum* NF54 strain in *An. gambiae* or *Anopheles stephensi* [8] or with field isolates of *P. falciparum* in *An. gambiae* [71]. This suggests more complex interaction of ivermectin on mosquito and parasite interaction, possibly by influencing mosquito midgut microbiota. The mosquito midgut microbiota composition has recently been shown to dramatically alter *Anopheles* immune response and thus *Plasmodium* infection [72]. Original investigations of avermectin, the biological precursor of ivermectin, suggested no direct effect on a range of bacteria species [73]. However, more recent evidence indicates that avermectin can inhibit growth of *Staphylococcus aureus* [74], and ivermectin can inhibit growth of *Chlamydia trachomatis* [75] and *Mycobacterium tuberculosis* [76]. This recent antibacterial evidence suggests that ivermectin may influence mosquito midgut microbiota composition, which could in turn alter *Plasmodium* infection. Recently, the midgut microbiota composition was shown to significantly alter formation of the peritrophic matrix [77], thus possible alteration of the midgut microbiota by ivermectin may influence formation of the peritrophic matrix and in turn alter *Plasmodium* infection. Since different insectaries around the world likely have different midgut bacteria microbiota compositions in colonized mosquitoes, this may explain the differences observed by different laboratories when investigating ivermectin sporontocidal effects. Much remains to be explored to determine the sporontocidal mode of action of ivermectin.

Concomitant ingestion of *P. vivax* and ivermectin LC_25_ failed to alter *An. dirus* survivorship compared to ivermectin LC_25_ alone (Fig. 8). This in contrast to the finding that *P. falciparum*-infectious *An. gambiae* that ingested ivermectin LC_25_ at DPI 14 were significantly, albeit modestly, more susceptible to ivermectin compared to uninfected *An. gambiae* [13]. There were some caveats to the *P. vivax* and *An. dirus* survival study in that mosquitoes had to be transported between and housed in two different insectaries and *P. vivax* blood was collected freshly from a single donor in sodium heparin tubes and uninfected blood from multiple donors was stored briefly in CPDA-1 bags. There was no significant difference in *An. dirus* survivorship when fed a control or ivermectin LC_25_ meal mixed with blood collected in CPDA-1 or sodium heparin tubes (Additional file 1: Figure S3, additional information text). Future investigations are warranted to determine whether *Plasmodium* infection in *Anopheles* alters susceptibility to ivermectin.

During ivermectin MDAs for malaria, numerous neglected tropical diseases (NTDs) in the GMS would be affected, including lymphatic filariasis, scabies, lice, gnathostomiasis, and soil-transmitted helminths (STHs) such as strongyloidiasis, ascariasis and trichuriasis. It has been estimated that 50% of persons in resource-poor communities in the GMS have one or more STH [78], and strongyloidiasis afflicts between 40 to 60% of persons in rural Cambodia [79] and Laos [80]. Ivermectin was found to be very effective at treating strongyloidiasis and repeated MDAs would further benefit afflicted communities as re-infection rates in Cambodia can be quite high [81]. Years of experience with ivermectin MDA for onchocerciasis in Africa have demonstrated that treated persons clearly recognize and appreciate the secondary benefits that ivermectin treatment has on NTDs [82].

MDA with artemisinin combination therapy (ACT) is being piloted in the GMS [1] and Africa [83]. One problem facing ACT MDA is that asymptomatic persons may not perceive a direct personal benefit of clearing malaria parasites. However, if ivermectin were incorporated into ACT MDA then this may improve compliance as persons could observe a direct personal benefit to MDA participation by reducing NTD burdens. Ivermectin and ACT MDA for malaria control, when rationally deployed in targeted hotspot areas of active *Plasmodium* transmission, would act in concert by clearing infected persons of their malaria parasites while reducing the transmission potential of the extant mosquito population. This could essentially reset malaria transmission when the next wave of naïve *Anopheles* emerge from the larval habitat and feed on a population cleared of their malaria parasites. Modelling suggests that if ivermectin is added to anti-malarial drug MDA this will reduce the number of MDAs and time required to achieve elimination [84]. The combination of ivermectin and artemether-lumefantrine was shown to be very safe [8]. Two clinical trials are currently being conducted to assess the safety, tolerability, pharmacokinetics and mosquito-lethal efficacy of ivermectin and dihydroartemisinin-piperaquine in Thailand (NCT02568098) and Kenya (NCT02511353). If ivermectin can integrate with ACT MDA, then this could become a powerful new tool for malaria elimination.

## Conclusions

Key malaria vectors in the GMS are susceptible to ivermectin at human relevant concentrations. Ivermectin is sporontocidal to *P. vivax* in *An. dirus* and *An. minimus*. A population pharmacokinetic model was developed for ivermectin after a single oral dose administration of 200 µg/kg in healthy volunteers. The developed model described observed data adequately and was used to simulate population mean concentration–time profiles after 200, 400 and 800 µg/kg doses of ivermectin. Mosquito in vitro survivorship results and pharmacokinetic model results indicate that the ivermectin dose of 400 µg/kg is the ideal minimal dose to administer during MDA in the GMS. There may be active ivermectin metabolites that extend the duration of mosquito-lethal effect beyond what is predicted by parent compound concentrations. Ivermectin MDA could be a powerful new tool to combat outdoor malaria transmission and assist malaria elimination efforts in the GMS.

## Additional file

**Additional file 1.** Supplemental information for pharmacokinetic model goodness of fit, mosquito infection and mosquito survival output.

## Abbreviations

ACT: artemisinin combination therapy
BMI: body mass index
CPDA: citrate phosphate dextrose adenine
DMSO: dimethylsulfoxide
DPI: day post infection
GMS: Greater Mekong Sub-region
LC_50_: lethal concentration that kills 50% of mosquitoes
MDA: mass drug administration
NTDs: neglected tropical diseases
OFV: objective function value
PBS: phosphate buffered saline
STHs: soil-transmitted helminths
