# Supplementary material for: Ivermectin susceptibility and sporontocidal effect in Greater Mekong Subregion Anopheles
Source: Malar J. 2017 Jul 7;16:280. doi: 10.1186/s12936-017-1923-8 (PMC5501099; doi:10.1186/s12936-017-1923-8)
Supplement: Supplementary file 1 — Additional file 1. Supplemental information for pharmacokinetic model goodness of fit, mosquito infection and mosquito survival output. [file 12936_2017_1923_MOESM1_ESM.docx]

**Additional Information Text**

**Anticoagulant effects on mosquito survival**

Blood was drawn from a single donor and put directly into 10 ml sodium heparin tubes (NH) (158 USP units, BD Vacutainer, Franklin Lakes, NJ, USA) or 6ml CPDA Vacuette tubes (Greiner Bio-One, Kremsmϋnster, Austria) under protocol (Protocol WRAIR#2079). It was then mixed with ivermectin LC_25_ or control and fed to *An. dirus*. Mosquito mortality was observed for seven days, any remaining mosquitoes at day 7 were counted as alive.

Anticoagulant type did not alter survivorship of *An. dirus* that ingested blood with ivermectin LC_25_ in either sodium heparin or CPDA (χ^2^=1.712, P=0.1907, reps=3, n=296) (Fig. S3). Anticoagulant type did not alter survivorship of *An. dirus* that ingested blood with control in either sodium heparin or CPDA (χ^2^=0.7067, P=0.4005, reps=2, n=195) (Fig. S3).

**Fig. S1. Goodness of fit of the final population pharmacokinetic model of ivermectin**

(A) observed concentrations vs population predictions, (B) observed concentrations vs individually predicted concentrations. Dashed lines represent the line of identity.

**Fig. S2 . *Plasmodium vivax* oocyst intensity in *An. dirus* when ivermectin LC_5_ ingested at DPI -3**

**Fig. S3. Survivorship of *An. dirus* when ivermectin LC_25_ control blood meal ingested when blood collected in sodium heparin or CPDA anticoagulants**

Survivorship between mosquito treatment groups was not significantly different as determined by the Mantel-Cox method.

**Table S1.** **Hazard of mosquito mortality post ivermectin blood meal at day 7 by species**

*An. dirus*

| Conc.* | IRR** | 95% CI*** | P-value |
| --- | --- | --- | --- |
| 100 | 64.3 | [29.9-138.3] | **<0.0001** |
| 95 | 58.1 | [27.0-124.9] | **<0.0001** |
| 85 | 55.7 | [25.7-120.8] | **<0.0001** |
| 80 | 46.8 | [22.0-99.3] | **<0.0001** |
| 70 | 42.5 | [20.0-90.3] | **<0.0001** |
| 65 | 44.5 | [20.8-95.2] | **<0.0001** |
| 60 | 36.1 | [16.9-76.9] | **<0.0001** |
| 55 | 29.6 | [13.9-63.2] | **<0.0001** |
| 50 | 32.3 | [15.2-68.7] | **<0.0001** |
| 45 | 21.4 | [10.0-45.8] | **<0.0001** |
| 40 | 19.1 | [8.8-41.7] | **<0.0001** |
| 35 | 24.4 | [11.1-53.3] | **<0.0001** |
| 30 | 4.7 | [1.9-11.3] | **0.001** |
| 25 | 8.2 | [3.4-19.4] | **<0.0001** |
| 20 | 2.8 | [1.1-6.9] | **0.027** |

*An. minimus*

| Conc. | IRR | 95% CI | P-value |
| --- | --- | --- | --- |
| 30 | 11.5 | [7.1-18.6] | **<0.0001** |
| 28 | 10.5 | [6.5-16.9] | **<0.0001** |
| 25 | 9.0 | [5.5-14.7] | **<0.0001** |
| 20 | 9.5 | [5.9-15.2] | **<0.0001** |
| 18 | 7.0 | [4.4-11.2] | **<0.0001** |
| 15 | 5.8 | [3.5-9.6] | **<0.0001** |
| 12 | 4.6 | [2.8-7.6] | **<0.0001** |
| 10 | 3.8 | [2.3-6.3] | **<0.0001** |
| 8 | 2.8 | [1.7-4.6] | **<0.0001** |
| 6 | 1.3 | [0.6-2.7] | 0.5150 |
| 5 | 3.6 | [2.0-6.2] | **<0.0001** |
| 4 | 2.9 | [1.7-5.0] | **<0.0001** |
| 3 | 1.8 | [0.9-3.9] | 0.1130 |
| 2 | 0.5 | [0.2-1.4] | 0.1770 |
| 1 | 2.7 | [1.3-5.7] | **0.0070** |

*An. sawadwongporni*

| Conc. | IRR | 95% CI | P-value |
| --- | --- | --- | --- |
| 40 | 9.4 | [5.5-16.2] | **<0.0001** |
| 35 | 8.4 | [4.9-14.6] | **<0.0001** |
| 30 | 8.0 | [4.8-13.4] | **<0.0001** |
| 28 | 5.2 | [3.0-9.1] | **<0.0001** |
| 25 | 5.3 | [3.1-9.0] | **<0.0001** |
| 22 | 2.3 | [1.3-4.1] | **0.004** |
| 20 | 2.7 | [1.5-4.8] | **0.001** |
| 18 | 2.1 | [1.1-4.0] | **0.031** |
| 15 | 2.1 | [1.2-3.8] | **0.012** |
| 12 | 0.9 | [0.3-2.7] | 0.896 |
| 10 | 1.1 | [0.5-2.5] | 0.829 |
| 8 | 0.6 | [0.2-2.2] | 0.472 |
| 6 | 0.3 | [0.0-2.0] | 0.194 |
| 5 | 1.3 | [0.5-3.3] | 0.573 |
| 4 | 0.3 | [0.0-2.4] | 0.271 |

*An. campestris*

| Conc. | IRR | 95% CI | P-value |
| --- | --- | --- | --- |
| 50 | 5.3 | [3.8-7.2] | **<0.0001** |
| 45 | 4.9 | [3.5-7.0] | **<0.0001** |
| 35 | 2.5 | [1.3-4.8] | **0.005** |
| 30 | 2.5 | [1.7-3.6] | **<0.0001** |
| 25 | 3.4 | [2.5-4.6] | **<0.0001** |
| 22 | 3.4 | [2.4-4.8] | **<0.0001** |
| 20 | 2.5 | [1.8-3.5] | **<0.0001** |
| 18 | 2.7 | [1.9-4.0] | **<0.0001** |
| 15 | 1.8 | [1.3-2.6] | **0.002** |
| 10 | 1.0 | [0.6-1.5] | 0.883 |
| 8 | 1.2 | [0.7-1.9] | 0.465 |
| 6 | 1.0 | [0.5-2.0] | 0.916 |
| 5 | 1.4 | [0.9-2.3] | 0.166 |
| 4 | 1.7 | [1.1-2.6] | **0.022** |
| 3 | 0.8 | [0.3-1.8] | 0.552 |

*Conc. = concentration of ivermectin imbibed in ng/ml. **IRR = Incidence Rate Ratio of mortality at day 7 between each treatment group divided by the control group. *** 95%CI = 95% Confidence Intervals. Significant P-values (P <0.05) are in bold.
